# Eco-friendly highly efficient BN/rGO/TiO_2_ nanocomposite visible-light photocatalyst for phenol mineralization

**DOI:** 10.1007/s11356-021-15083-y

**Published:** 2021-07-02

**Authors:** Shekhah Al-Kandari, Aboubakr M. Abdullah, Halema Al-Kandari, Gheyath K. Nasrallah, Mohammed A. Sharaf, Douaa S. AlMarzouq, Ahmed M. Mohamed, Nadin Younes, Nada Kafour, Talal Al-Tahtamouni

**Affiliations:** 1 Chemistry Department, Faculty of Science, Kuwait University, P.O. Box 5969 Safat, 13060 Kuwait City, Kuwait; 2 Center for Advanced Materials, Qatar University, Doha, P.O. Box 2713, Qatar; 3 Department of Health Environment, College of Health Sciences, PAAET, P.O. Box 1428, Faiha, 72853 Kuwait City, Kuwait; 4 Department of Biomedical Science, College of Health Sciences, QU Health, Qatar University, Doha, P.O. Box 2713, Qatar; 5 Biomedical Research Center, Qatar University, Doha, P.O. Box 2713, Qatar; 6 Department of Maritime Transportation Management Engineering, İstanbul University-Cerrahpaşa, Avcilar, 34320 Istanbul, Turkey; 7 Materials Science and Technology Program, College of Arts and Sciences, Qatar University, Doha, P.O. Box 2713, Qatar

**Keywords:** Boron nitride, Reduced graphene oxide, TiO_2_, Photocatalysis, Phenol degradation, Ecotoxicology, Zebrafish embryo model

## Abstract

Boron nitride (BN) and reduced graphene oxide (rGO) of different loadings were composited with commercial P25 TiO_2_ (Ti) through the hydrothermal method. The as-prepared nanocomposites were characterized using various techniques: X-ray photoelectron spectroscopy, X-ray diffraction, thermal gravimetric analysis, Fourier transform infrared and Raman spectroscopies, and transmission and scanning electron microscopies. It was observed that 10% and 0.1% of BN and rGO, respectively, loaded on TiO_2_ (10BNr0.1GOTi) resulted in the best nanocomposite in terms of phenol degradation under simulated sunlight. A 93.4% degradation of phenol was obtained within 30 min in the presence of H_2_O_2_. Finally, to ensure the safe use of BNrGOTi nanoparticles in the aquatic environment, acute zebrafish toxicity (acutoxicity) assays were studied. The 96-h acute toxicity assays using the zebrafish embryo model revealed that the LC_50_ for the BNrGOTi nanoparticle was 677.8 mg L^−1^ and the no observed effect concentration (NOEC) was 150 mg L^−1^. Therefore, based on the LC_50_ value and according to the Fish and Wildlife Service Acute Toxicity Rating Scale, BNrGOTi is categorized as a “practically not toxic” photocatalyst for water treatment.

## Introduction

Photocatalysis is gaining more attention in water pollution management in which refractory pollutants are ultimately converted into H_2_O and CO_2_ (Satpal and Athawale 2018). The phenolic compounds, including phenol, are confirmed in various wastewaters. They are listed by the United States Environmental Protection Agency (US-EPA) as prior compounds (Ahmed et al. 2010). Phenol is a refractory compound to the traditional water treatment technologies, and its concentration should satisfy the World Health Organization (WHO) standards before dumping it into the aquatic environment (Akbal and Nur Onar 2003, Yan et al. 2006, Tao et al. 2013, Abdullah, Al-Thani, et al. 2016). Despite titanium dioxide, which is extensively tested as a photocatalyst semiconductor, its industrial application is restricted due to its high bandgap energy, which means the utilization of sunlight as an excitation source is limited to its UV component. Numerous research works demonstrated that compositing TiO_2_ with an additional semiconductor and/or carbon material improved the photocatalytic activity of TiO_2_. Graphene and hexagonal boron nitride (h-BN) are two-dimensional materials arranged on a honeycomb structure. These two materials attracted much attention due to their exceptional electronic features (Wei et al. 2011, Liu et al. 2017). We proved, earlier, that a photocatalyst from TiO_2_ and reduced graphene oxide (rGO) (Al-Kandari et al. 2015a, 2015b, Abdullah et al. 2016, Al-Kandari, Abdullah et al. 2016, Al-Kandari, Abdullah et al. 2016, Al-Kandari et al. 2017a, 2017b) degrades a variety of organic compounds efficiently under simulated sunlight. In this respect, we found that 0.1% of rGO with TiO_2_ yielded 63% of phenol degradation within 30 min in the presence of an eco-oxidant, H_2_O_2_ (Al-Kandari et al. 2017a, 2017b). Wang et al. (2017) mentioned and reviewed in their article different preparation methods of BNrGO and their applications in nanoelectronic devices; however, they did not report any study for water treatment.

In this research work, novel composites (BNrGOTi) consisting of (2% or 10%) BN, (0.01% or 0.1%) rGO, and commercial P25 TiO_2_ were synthesized using a hydrothermal treatment, characterized using different techniques, and evaluated for phenol degradation under simulating sunlight. In order to ensure the safe use of BNrGOTi nanoparticles in the aquatic environment, zebrafish acute toxicity (acutoxicity) assays were used. Zebrafish is an invaluable and reliable aquatic model that is increasingly used for measuring drug and nanoparticle toxicity (Zakaria et al. 2018).

## Materials and methods

### Chemicals

Boron nitride (BN) nanomaterials (40 nm and 99.9% purity), titanium dioxide P25 (21-nm particle size), and hydrogen peroxide (30%) were used for the preparation of nanocomposites. Diethylaminobenzaldehyde (DEAB) was used as a positive control (PC) in the acutoxicity assays due to its nature to cause toxic teratogenic effects on zebrafish models. N-phenylthiourea (PTU) in egg water (also known as E3 medium), PTU-E3 medium, was used to raise the zebrafish embryos in vitro. PTU was used to inhibit the formation of pigmentation in the growing embryos, allowing us to facilitate imaging under the microscope. The E3 media used consist of sodium chloride (NaCl), potassium chloride (KCl), calcium chloride dihydrate (CaCl_2_·2H_2_O), and magnesium sulfate heptahydrate (MgSO_4_·7H_2_O). Stock solutions, including egg water, PBS, PTU, and methylene blue, are prepared according to protocols described in previous studies (Nasrallah et al. 2018, Rasool et al. 2018, Younes et al. 2018). In order to prepare working solutions, a purified Milli-Q water (Millipore, France) was used. The nanoparticle stock solutions were prepared by adding 0.02 mg of the nanoparticle to 10 mL of 1× PTU-E3 media. In order to properly dissolve the nanoparticle in the media, the stock solution was probe sonicated twice for 5 min. A freshly prepared stock solution is then rediluted using PTU-E3 media to reach the required concentrations of 50, 100, 150, 200, and 250 mg L^−1^. All chemicals mentioned previously were purchased from Sigma-Aldrich, Steinheim, Germany.

### Preparation of TiO_2_-supported BN and rGO

Graphene oxide (GO) was prepared using the modified Hummers’ method, as mentioned earlier (Al-Kandari et al. 2015a, 2015b). TiO_2_-supported BN and rGO (BNrGOTi) nanocomposites of different concentrations were prepared using hydrothermal treatment as follows: TiO_2_ was added to a mixture of deionized water/absolute ethanol (1:1 ratio) and sonicated for 30 min. The same procedure was applied for BN and GO, each separately. After that, the three suspensions were mixed and stirred for 30 min, then shifted to a Teflon-lined stainless steel autoclave at 120°C overnight. Lastly, the suspension was dried in an oven at 80°C for 24 h. The as-prepared composites were abbreviated as XBNYrGOTi, where X and y are allocated for the percentage of BN and rGO, respectively, loaded in TiO_2_ (Ti).

### Materials characterization

The morphology and composition of the as-synthesized composites were characterized using a Hitachi S-4800 (Hitachi, Tokyo, Japan) scanning electron microscope (SEM) equipped with an energy-dispersive spectrometer (EDS) and a TecnaiG220 (FEI, Hillsboro, OR, USA) transmission electron microscope (TEM). X-ray photoelectron spectroscopy (XPS) was carried out with a Kratos Axis (Ultra DLD XPS Kratos, Manchester UK) equipped with a monochromatic Al Kα radiation source (1486.6 eV) under an ultra-high vacuum environment (approximately 5 × 10^−9^ Torr). The BET surface area was measured using a Quantachrome Autosorb-1 analyzer (Quantachrome Instrument Corporation, Boynton Beach, FL, USA). The X-ray diffraction (XRD) patterns were recorded using an X-ray diffractometer (X’Pert-Pro MPD, PANalytical Co., Almelo, The Netherlands) with a Cu Kα X-ray source (λ = 1.540598 Å). The Fourier transform infrared and Raman spectra were recorded on a Thermo Nicolet Nexus 670 FT-IR spectrometer (Thermo Scientific, Madison, WI, USA) and PerkinElmer RamanStation 400 spectrometer with a 532-nm laser as an excitation source. More details about the characterization techniques used can be found elsewhere (Al-Kandari et al. 2017a, 2017b). The optical band gap (ultraviolet–visible diffuse reflectance spectroscopy [UV-Vis DRS]) was measured using a Cary 5000 UV-Vis-NIR spectrophotometer (Agilent, Austria) equipped with an integrating sphere accessory. The BET surface areas were measured using an automatic ASAP 2010 MICROMERITICS sorpometer (USA) outfitted with an outgassing platform and online data acquisition and handling system operating at various computer-run methods.

### Photocatalytic experiments

The representation of the photocatalytic reaction arrangement was declared elsewhere (Al-Kandari et al. 2016a, 2016b). In each run, 100 mL of phenol solution of 15 ppm was used. The suspension of 0.1-g catalyst and phenol solution was stirred without pH adjustment in a dark chamber for 30 min to reach adsorption equilibrium. Next, a 150 W Xe lamp as an excitation source was turned on ( time equals zero) without a cut-off filter at an integrated intensity of 12 mW cm^−2^. The samples were drawn from the reaction vessel every 5 min and filtered using a nylon filter paper of pore size 0.4 μm. It is good to note that no phenol elimination was observed using the mentioned nylon filter paper, as demonstrated earlier (Al-Kandari et al. 2018). The progress of the photocatalytic reaction was monitored using a UV**-**Vis spectrophotometer in the range of 190–400 nm, with 279 nm corresponding to the highest absorption of phenol. The percent of phenol degradation was calculated using the following equation:

1 $$ \mathrm{Degradation}\%=\left[\frac{\left({C}_0-{C}_t\right)}{C_0}\right]\times 100 $$

*C*_0_ is the concentration of phenol before the Xe lamp was turned on, while Ct designated the remaining phenol concentration after irradiation for a certain time *t*. The total organic carbon (TOC) for all filtrates was measured using a TOC-VPH Shimadzu analyzer (Kyoto, Japan).

### Zebrafish culture and acute toxicity assays

Throughout the study, the zebrafish AB strain was used to carry out the acute toxicity experiments. For a more detailed insight on our aquatic zebrafish system (Aquaneering, CA, USA), source, culture, maintenance, and mating protocol of the zebrafish lab, the reader is advised to read the following articles (Nasrallah et al. 2018, Rasool et al. 2018, Younes et al. 2018, Abou-Saleh et al. 2019, Nasrallah et al. 2019). When carrying out the acute toxicity assay, zebrafish embryos were collected at 24 h post-fertilization (hpf) and dechorionated following a previous study (Abou-Saleh et al. 2019). The dechorionation process involved removing the preexisting E3-media from the plate and the addition of 0.5 mg/mL of pronase enzyme (Sigma, Germany) in 10-mL PTU-E3 media. The plate was then incubated for 7 min at 28°C in order to allow the chorion to soften and then the media was washed out 2–3 times and replaced with PTU-E3 media. Screening of the plate was then carried out under the standard stereomicroscope (Zeiss, Germany) to remove unhealthy or abnormal embryos.

### Acutoxicity assays (LC_50_ and NOEC)

The selected healthy embryos (from the previous experiment) were then placed in a 12-multiwell plate; 15 embryos in each well containing (i) 5 mL of PTU-E3 media as a negative control (NC), (ii) 5 mL of five different concentrations of BNrGOTi (50, 100, 150, 200, 250 mg L^−1^), and (iii) three concentrations of DEAB as a positive control (PC) (1, 10, 100 μM). The plate is then incubated at 28°C for an additional 72 h in order for the embryos to reach the desired 96 hpf for imaging. The survival rate was recorded every 24 h throughout the experiment. After 96 hpf, the survival rate was calculated for each group by counting the number of dead embryos over the number of live embryos as a percentage. If the embryos were observed to have coagulation of unfertilized eggs, no somite formation, lack of detachment of tail bud from the yolk sac, and no heartbeat, these embryos were counted as dead. The sigmoidal mortality curve was plotted using the GraphPad Prism 7 software, and a lethal concentration of 50 (LC_50_) was then calculated (Nasrallah et al. 2018). To calculate the no observed effect concentration (NOEC), the common body deformities (teratogenicity) were examined at 96 hpf compared to the negative and positive controls. The body deformities, including the size of the yolk (yolk edema), the heart (heart edema), and the eye in addition to body length or scoliosis, were imaged, and the sizes were scored using the ImageJ software as explained elsewhere (Younes et al. 2018, Abou-Saleh et al. 2019, Al-Kandari et al. 2019, Nasrallah et al. 2019, Younes et al. 2019). Cardiotoxicity was assessed by measuring the heart rate in the dorsal aorta and pericardial vein (PCV) using the MicroZebraLab blood flow from Viewpoint (version 3.4.4, Lyon, France) as described in Al-Asmakh et al. (2020) and Al-Jamal et al. (2020). The NOEC is the highest concentration used in the experiment that had no significant (<20%) mortality or teratogenicity or cardiotoxicity compared to the negative control.

## Results and discussion

### Characterization

**Fig. 1:**
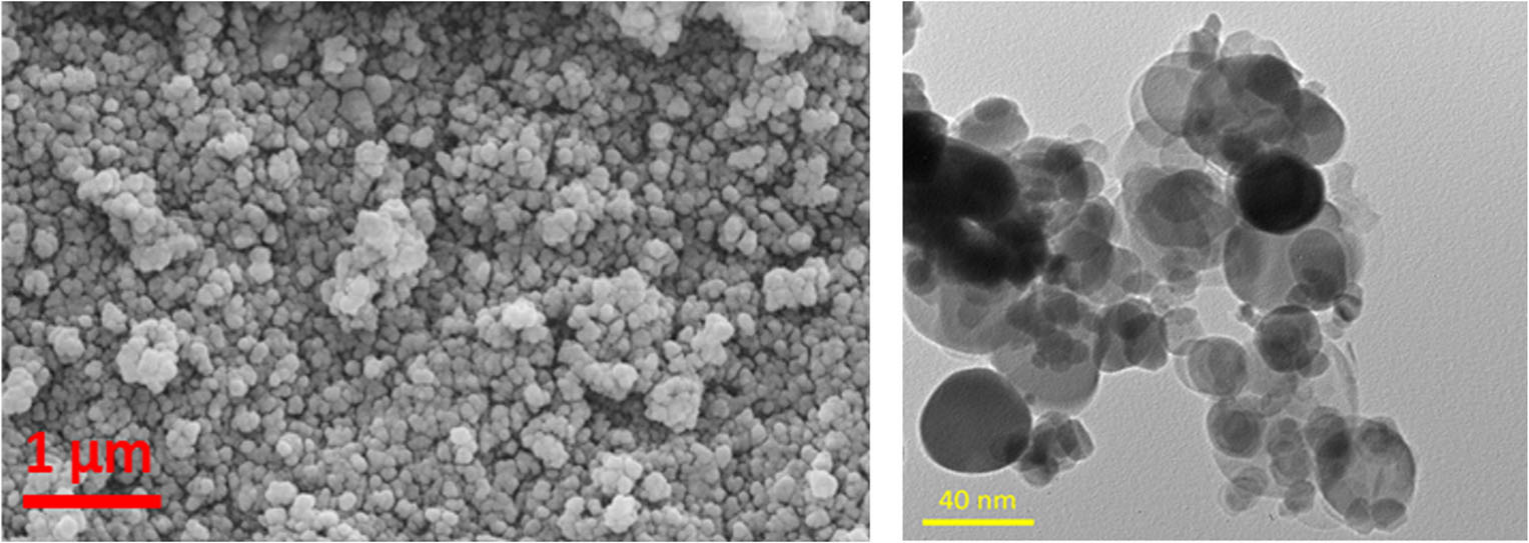
SEM (left) and HRTEM (right) micrographs for BNrGOTi nanocomposite

Figure 1 shows the SEM and high-resolution transmission electron microscopy (HRTEM) micrographs for the nanocomposite. Two different particle sizes can be detected, one around 20 nm for the TiO_2_ and almost double for the BN. The rGO was not detected from the images, but the EDS analysis confirmed B, N, O, C, and Ti. Also, Raman spectroscopy, as will be shown later, confirmed its existence. This may be attributed to the small loading of rGO (0.1%).

**Fig. 2:**
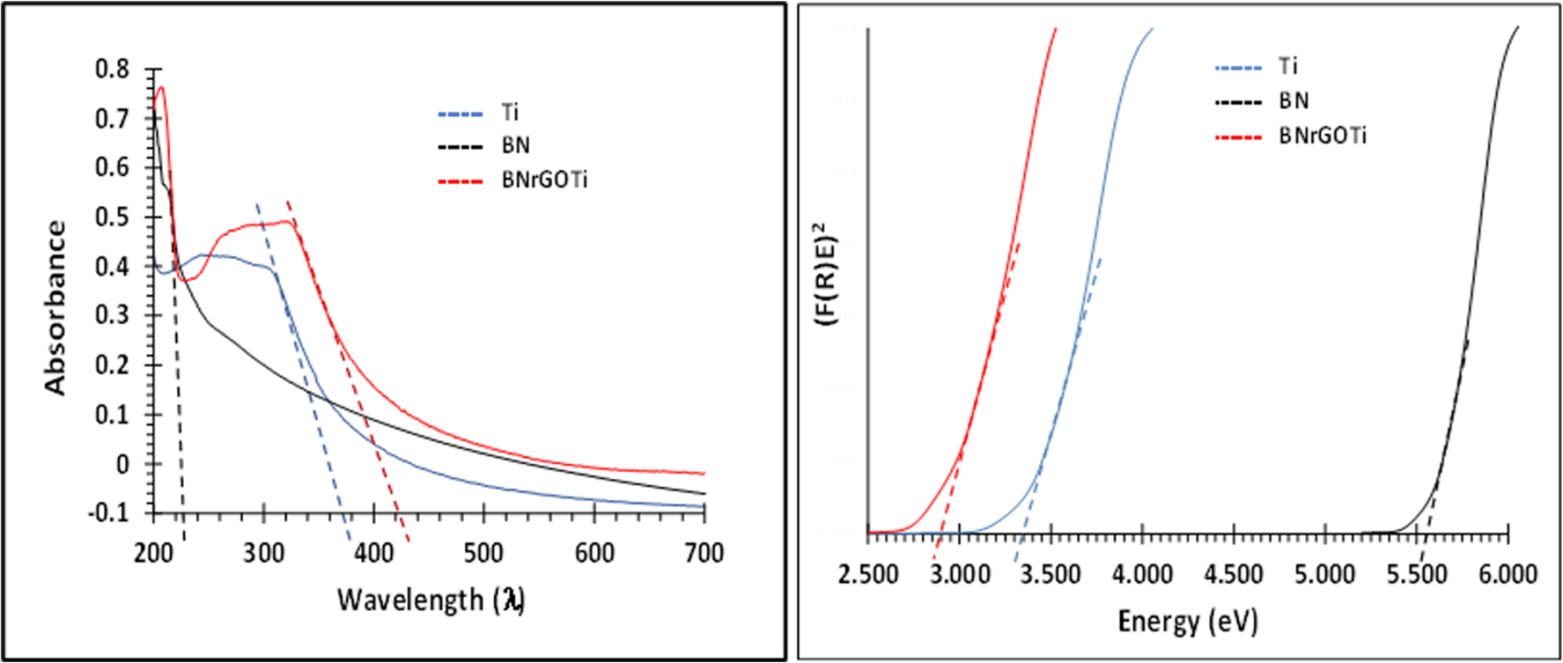
The UV spectra and bandgap energies of TiO_2_, BN, and BNrGOTi

The UV diffuse reflectance was performed for BN, TiO_2_, and BNrGOTi to identify their bandgap energy corresponding to the wavelengths at the absorption edge (Fig. 2). The bandgap energy for BN is 5.55 eV, corresponding to 223.39 nm. It can be seen that a significant reduction of the bandgap energy of TiO_2_ after compositing it with BN and rGO from 3.35 (370.10 nm) to 2.94 eV (421.71 nm), i.e., the photocatalytic activity of TiO_2_ is shifted from the UV to the visible region after compositing it with the rGO and BN.

**Fig. 3:**
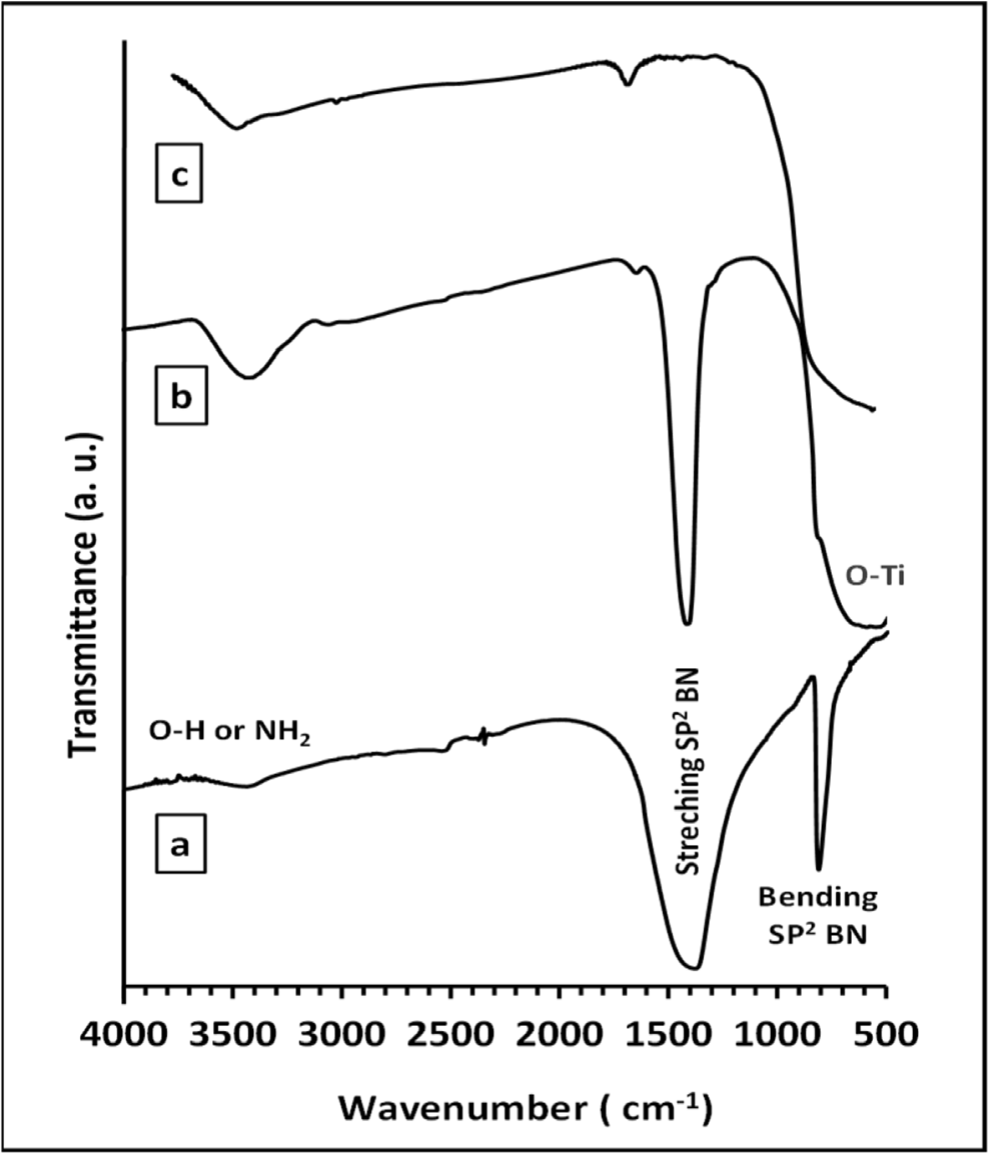
FT-IR for **a** pure BN, **b** BNrGOTi, and **c** pure TiO_2_

Figure 3 shows the FT-IR spectra for pure BN, TiO_2_, and the as-prepared composite BNrGOTi. Two typical absorption bands of hexagonal BN (h-BN) were noticed at 1378 and 810 cm^−1^ related to stretching and bending modes of sp^2^ hybridized BN skeletons, respectively (Zheng et al. 2007, Weng et al. 2015, Štengl et al. 2016, Sun and Xiao 2017, Qu et al. 2018). In addition, a broad band in the range of 3700–3300 cm^−1^ can be designated for O-H from the adsorbed water molecules on the surface of the sample and/or NH_2_ groups. In Fig. 3c, a strong and broad absorption band at a low frequency below 1000 cm^−1^, which is attributed to the Ti- O-Ti vibration in TiO_2_ (Al-Kandari et al. 2015a, 2015b). A peak was identified at 1627 cm^−1^, assigned for the deformed water molecules or Ti-O-Ti starching vibration. Additionally, a broad band in the range of 3800–3000 cm^−1^ could be allocated for intercalated water molecule or/and O-H starching vibration of the C-OH group (from the carboxylic acid groups) (Al-Kandari et al. 2017a, 2017b). After loading BN and rGO on TiO_2_, the absorption bands characteristic for BN and Ti remain (note that peaks at 806 cm^−1^ bears as a shoulder) with no observed distinctive peak at 1566 cm^−1^ for skeletal vibration of reduced graphene oxide (Al-Kandari et al. 2014; Al-Kandari et al. 2016a, 2016b). This may be due to the low loading of rGO (0.1%) in the as-prepared composite.

**Fig. 4:**
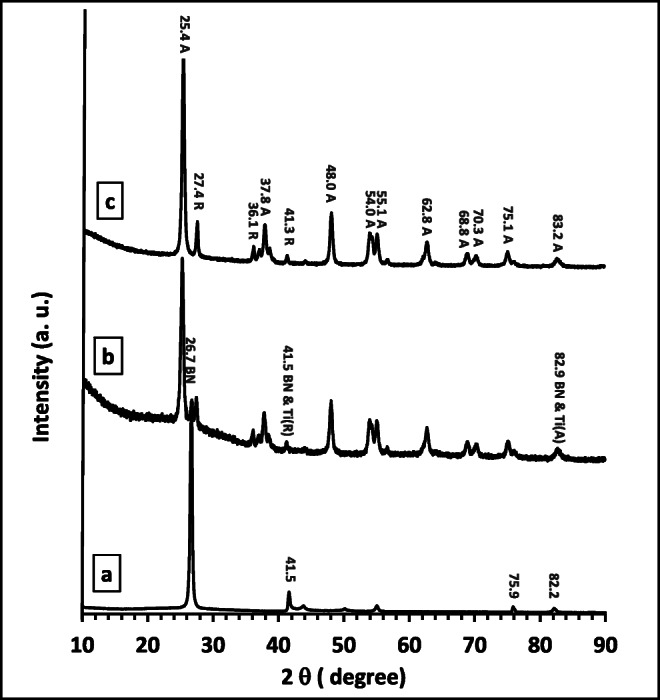
XRD spectra for **a** BN, **b** BNrGOTi, and **c** TiO_2_

X-ray diffraction patterns of BN, BNTi, and TiO_2_ were demonstrated in Fig. 4. It can be concluded that BN exists in a hexagonal shape with characteristic peaks at 26.7, 41.5, 43.8, 55.1, 75.9, and 82.2° (ICDD: 00-045-0896, ICDD: 98-002-4644). The main characteristic peaks at 2θ = 26.7 and 41.5° corresponding to 002 and 100 planes, respectively (Zheng et al. 2007, Liu et al. 2017, Singh et al. 2017, Sun and Xiao 2017). The XRD diffraction pattern of TiO_2_ (Fig. 4c) showed anatase and rutile phases of TiO_2_. The main characteristic peak anatase phase of TiO_2_ located at 2θ = 25.4° linked with the other peaks located at 37.8, 38.6, 48.0, 54.0, 55.1, 62.7, 68.8, 70.3, 75.1, 76.0, and 83.2° (ICDD: 98-017-2914). While the peak located at 2θ = 27.4° is the main peak for the rutile phase of TiO_2_ associated with other peaks located at 36.1, 39.2, 41.3, 44.1, 54.3, 62.7, 64.1, and 89.6° (ICDD: 00-021-1276, ICDD: 98-005-1935) (Al-Kandari et al. 2017a, 2017b). The XRD diffraction patterns of BNrGOTiO_2_ (Fig. 4b) exhibited the main peaks of h-BN besides the characteristic peaks of the tetragonal anatase, and rutile phase of TiO_2_ and without any peak at 2θ = 23.9° corresponding to reduced graphene oxide was observed (Al-Kandari et al. 2016a, 2016b). This is maybe due to the low loading of the reduced graphene oxide in the composite.

**Fig. 5:**
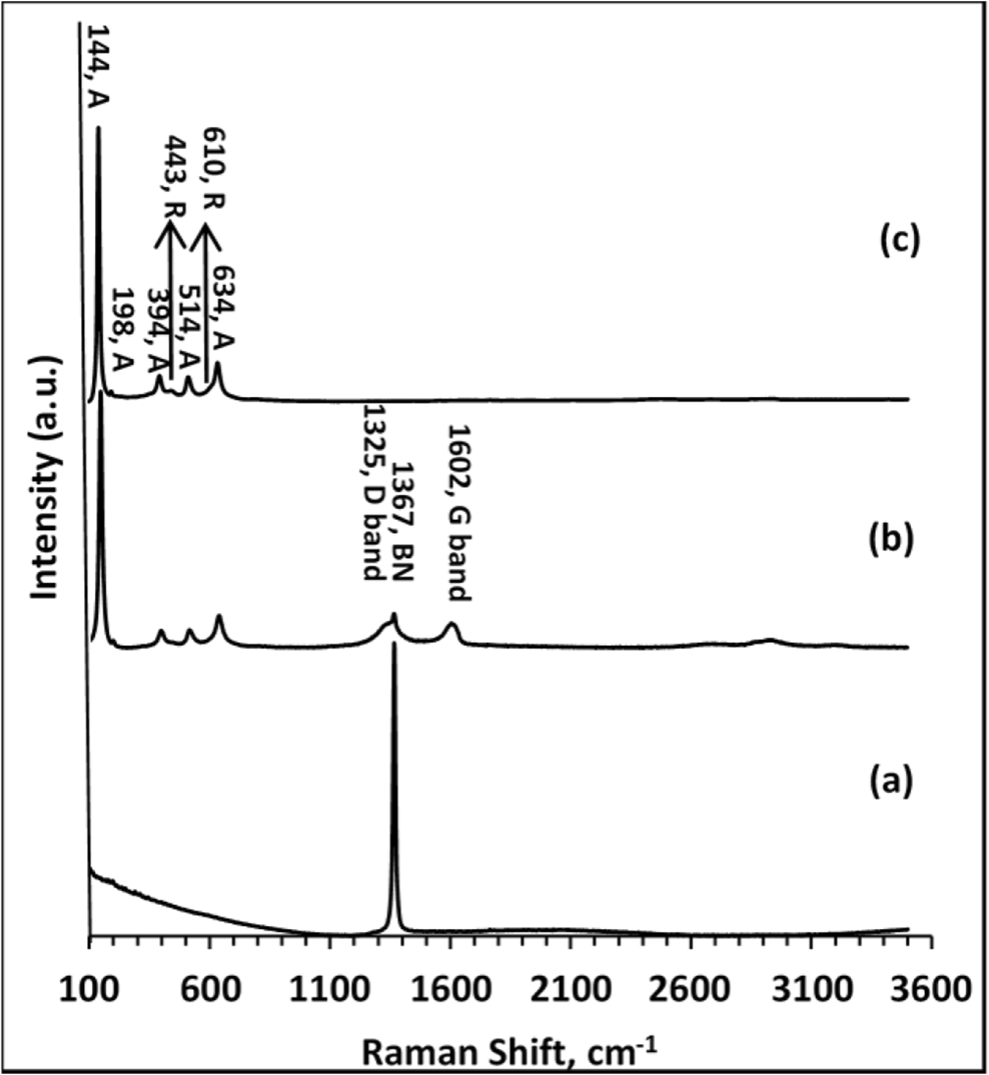
Raman spectra for **a** BN, **b** BNrGOTi, and **c** TiO_2_

Raman spectra were recorded for BN, TiO_2_, and BNrGOTi in the range of 100–3600 cm^−1^ at an excitation of 523 nm, as depicted in Fig. 5. The Raman spectrum of bare BN showed a strong characteristic peak of E2g vibration mode at 1367 cm^−1^ (Fig. 5a) (Weng et al. 2013, Nasr et al. 2017). The Raman spectra for TiO_2_ (Fig. 5c) identified vibration modes for both anatase and rutile phases of TiO_2_ located at 144 (Eg), 198 (Eg), 394 (B1g), 514 (B1g + A1g), and 634 (Eg) cm^−1^ the for anatase phase and a weak band at 443 cm^−1^ and hump at 610 cm^−1^ for the rutile phase. The BNrGOTi spectrum (Fig. 5b) showed the characteristic peaks for both BN and TiO_2_ besides the characteristic D and G bands for reduced graphene oxide located at 1325 and 1602 cm^−1^ (Al-Kandari et al. 2014; Al-Kandari et al. 2015a, 2015b; Al-Kandari et al. 2016a, 2016b; Al-Kandari et al. 2017a, 2017b).

**Fig. 6:**
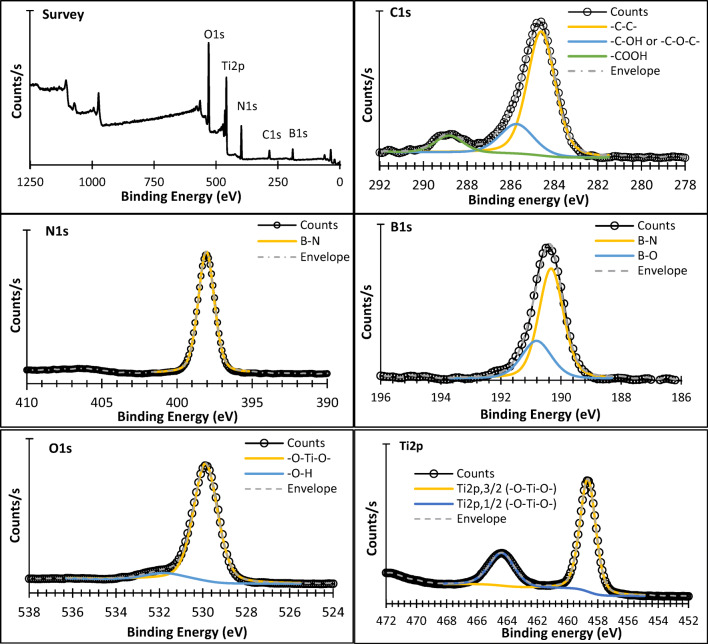
XPS survey, C1s, N1s, B1s, O1s, and Ti2p spectra of BNrGOTi

XPS results of BNrGOTi nanocomposite were given in Fig. 6 and Table 1. The survey spectrum showed N1s, C1s, B1s, Ti2p, and O1s on the surface of the composite. The deconvolution of the C1s band revealed the presence of C-C and C=C bonds at 248.60 eV, C-OH and/or -C-O-C- at 285.73 eV, and -COOH at 288.82 eV (Al-Kandari et al. 2015a, 2015b). The XPS narrow scan of the N1s region at 390 to 410 eV showed the presence of N1s at 398.05 eV. This result proves that nitrogen atoms are bonded to B atoms (B-N) (Qu et al. 2018) without any interaction with TiO_2_. The B1s region showed a main peak at 190.33 eV besides a small peak at 190.82 eV. The major peak at 190.33 eV is due to the B-N bond (Liu et al. 2017, Qu et al. 2018), while the peak of the small is mostly due to the B-O-Ti (Liu et al. 2017). Two peaks in the O1s region were observed at 529.88 and 531.79 eV. The former peak is due to the -O-Ti-O- and/or B-O-Ti bond, while the latter could be assigned for the -OH (Hasan et al. 2018). The Ti2P region showed only Ti2p, 3/2, and Ti2p, 1/2 at 458.66 and 464.35 eV, respectively, with a spacing of 5.69 eV. This result confirms titanium in oxidation state IV and no bond formation between either B or N with Ti.

**Table 1:** XPS results of BNrGOTi

Elements	Binding energy, eV	Chemical state	Atomic %	Total atomic %
O	529.88	-O-Ti-O- (O1s)	58.14	60.13
	531.79	-OH (O1s)	1.99	
Ti	458.66	-O-Ti-O- (Ti2p, 3/2)	7.68	11.31
	464.35	-O-Ti-O- (Ti2p, 1/2)	3.63	
B	190.33	B-N (B1s)	8.04	11.38
	190.82	B-O (B1s)	3.34	
N	398.05	B-N (N1s)	10.69	10.69
C	248.60	-C-C- (C1s)	4.54	6.49
	285.73	-C-OH or -C-O-C- (C1s)	1.31	
	288.82	-COOH (C1s)	0.64	

The BET surface areas of materials used in this study are summarized in Table 2. The surface areas of BN and graphite are 19.5 and 20.5 m^2^g^−1^, respectively. The surface area of graphite is increased from 20.0 to 40.0 m^2^g^−1^ during its oxidation to rGO. A tremendous increase in the surface area was observed when GO was reduced hydrothermally to reach 418.3 m^2^g^−1^. However, the BET surface areas of TiO_2_ and 10BNrGOTi are nearly the same. This leads to the conclusion that the BET surface is not the major factor for the photocatalytic activity of 10BN0.1rGOTi toward phenol degradation in this study.

**Table 2:** BET surface areas of materials used

Material	BET surface area (m^2^g^−1^)
BN	19.5
Graphite	20.5
GO	40.3
rGO	418.3
TiO_2_	51.1
BNrGOTi	50.3

### Photocatalytic reaction

**Fig. 7:**
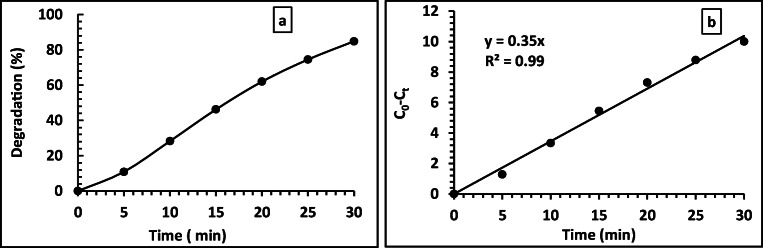
Degradation % (a) and rate of degradation (b) of 15 ppm phenol with time on 2BN0.1rGOTi under Xe illumination with 70 μL of H_2_O_2_

It was remarkable that no phenol degradation in the absence of the BNrGOTi and TiO_2_ photocatalysts, i.e., neither pure BN nor rGO or their composites have any catalytic activity toward phenol degradation. From previous studies, we revealed that 0.1% of rGO with TiO_2_ (0.1rGOTi) shifted the photoabsorption of TiO_2_ to the visible region and yielded the best catalytic activity toward phenol degradation (Al-Kandari et al. 2015a, 2015b, Al-Kandari, Abdullah et al. 2016, Al-Kandari, Abdullah, et al. 2017, Al-Kandari, Al-Kandari et al. 2020). Therefore, we were curious to see the effect of making a new composite of BN and rGO loaded on Ti on phenol degradation. The photocatalytic degradation of 15 ppm phenol on 2BN0.1rGOTi in the presence of 70 μL of H_2_O_2_ under a Xe illumination is demonstrated in Fig. 7. The phenol degradation rate was increased with time to reach 84.9 % in 30 min. It was noted that the phenol degradation in the composite followed pseudo-zero-order reaction (*C*_0_ − *C*_*t*_ = Kt) with linear regression (*R*^2^) equals 0.99 and rate equals 1.59 × 10^−4^ M min^−1^.

**Fig. 8:**
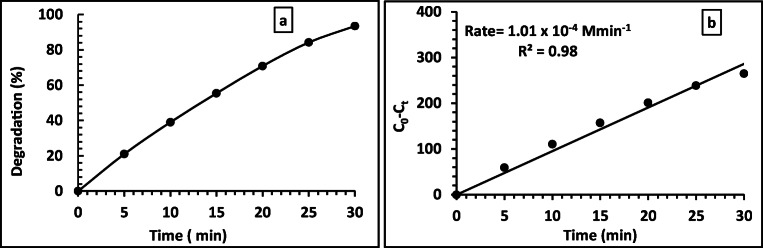
Transient degradation% (a) and rate of reaction (b) of 15 ppm phenol using 10BN0.1rGOTi under Xe illumination with 70 μL of H_2_O_2_

Looking for enhancing the phenol degradation rate, we increased the concentration of BN in the composite to 10%, while the concentration of rGO (0.1 %) was kept the same (Fig. 8).

**Fig. 9:**
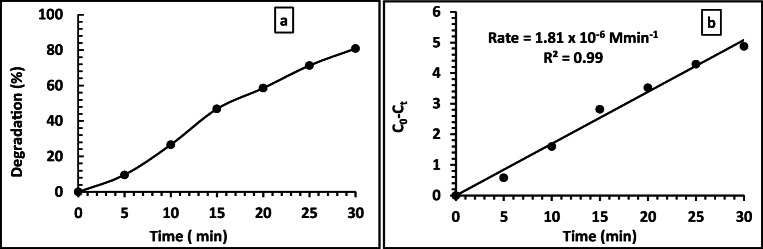
Transient degradation% (**a**) and rate of reaction (**b**) of 15 ppm phenol using 2BN0.1rGOTi under Xe illumination with 70 μL of H_2_O_2_

Also, another composite was prepared in which the concentration of rGO was reduced to 0.01, and the concentration of BN remained the same as the first composite (with 2% BN). The photocatalytic reaction was performed under the same condition, as shown in Fig. 8. We did not try to increase the concentration of rGO instead of decreasing it as we have done this in previous studies, but it did not show promising results [6, 14]. As shown in Fig. 8 and Fig. 9, the phenol degradation was increased from 84.9 to 93.4%, increasing the concentration of BN in the composite from 2 to 10%, respectively. While decreasing the concentration of rGO from 0.1 to 0.01% in the composite, a slight decrease in the phenol degradation was observed from 84.9 to 80.9. In both cases, the reaction rate followed well with pseudo-zero-order reaction with an excellent linear fit. From the previous studies, it can be concluded that 10BN0.1rGOTi is the best nanocomposite for phenol degradation under study. The TOC analysis has shown that 92% of the degraded phenol was completely mineralized to CO_2_ and water using the 10BN0.1rGOTi, which is lowered to 87% for the composite with lower BN content.

From the previous studies, it is observed that the phenol degradation rate improved sharply in the case of BNrGOTi under Xe illumination. This is due to the decrease in the bandgap energy of TiO_2_, so the wavelength of excitation is shifted from UV to the visible region. On the other hand, the huge delocalization of BN and rGO and easy transfer of electrons inhibited the rate of electron-hole pair recombination; therefore, the phenol degradation is improved dramatically.

### Ti-BN-rGO is a “green” photocatalysts nanoparticle

**Fig. 10:**
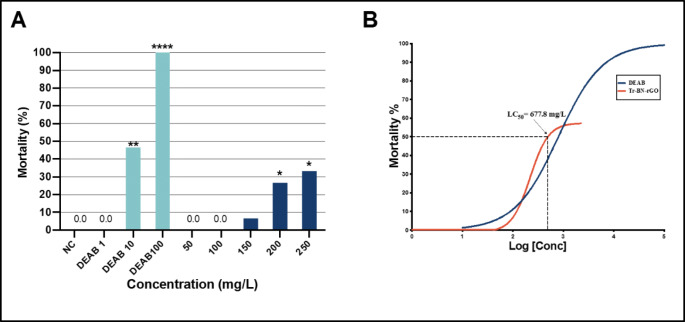
**A** Mortality/survival score at 96 hpf of embryos exposed to different concentrations of DEAB (positive control), NC (negative control), and 5 concentrations of BNrGOTi nanoparticles. **B** Logarithmic mortality response (LC_50_) curve for different concentrations of DEAB TN-BN-rGO nanoparticles. A chi-square test was used to compare the differences between the mortality rate between groups. *p < 0.05, **p < 0.01, and ***p < 0.001. Fifteen embryos were used per concentration (*n* = 15)

We examined the potential adverse effect of BNrGOTi nanocomposites on the zebrafish by utilizing the LC_50_, which is the most important and common acutoxicity parameter usually used in zebrafish acutoxicity experiments. As shown in Fig. 10A, the concentration of cumulative survival with no significant mortality or (<20%) for the BNrGOTi (93.3%) was 150 mg L^−1^, while for the DEAB positive control was at 1.0 μM. Based on the logarithmic survival curve, the calculated LC_50_ value for the BNrGOTi was 677.8 mg L^−1^ (Fig. 10B). The concentrations (0, 50, 100, 250, 200, 250 mg L^−1^) of nanoparticle used for the determination of LC_50_ were chosen based on significant environmental relevancy, as they all fall within the ranges of the Fish and Wildlife Service Acute Toxicity Rating Scale (Nasrallah et al. 2018). This rating scale categorizes any compounds’ toxicity based on LC_50_ value where 0.1–1.0 mg L^−1^ considered highly toxic, 1.0–10 mg L^−1^ moderately toxic, 10–100 mg L^−1^ slightly toxic, 100–1000 mg L^−1^ practically nontoxic, and >1000 mg L^−1^ is relatively harmless. Thus, based on the LC_50_ value and according to the Fish and Wildlife Service Acute Toxicity Rating Scale, BNrGOTi can be categorized as “practically not toxic” or “green” photocatalysts.

### BNrGOTi teratogenicity test

**Fig. 11:**
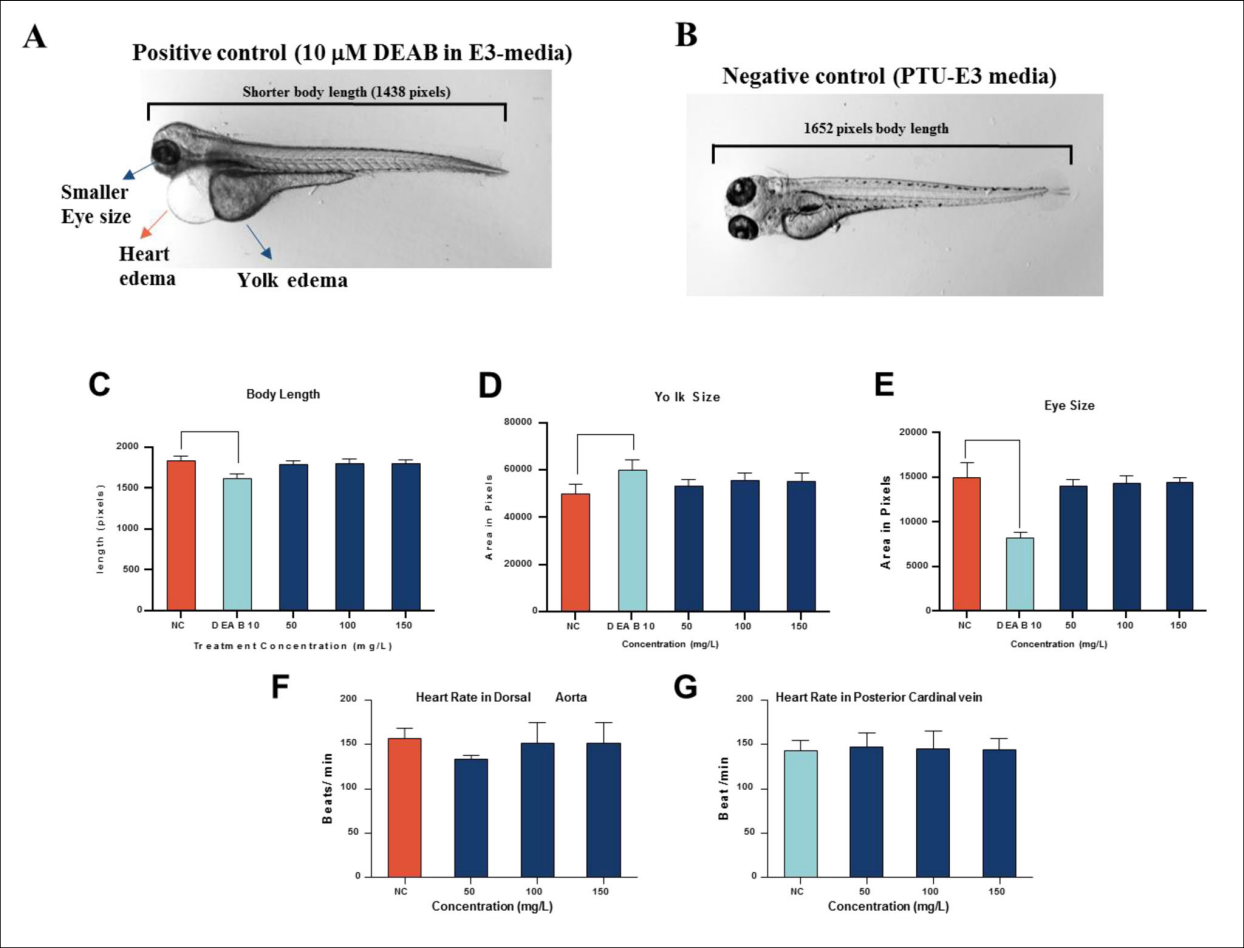
Images representing the effects of acute toxicity experiments of embryos exposed to **A** 10 μ DEAB as a positive control and **B** negative control PTU-E3 media only (96 hpf). Observed changes can be seen in the PC as heart edema, yolk edema, and a decrease in body length. Specific teratogenic changes analysis **C** average body length, **D** yolk size, and **C** eye size were measured using ImageJ software version 1.52a. Heart rate was calculated from the **E** dorsal aorta (DA) and **F** posterior cardinal vein (PCV) of the embryos following treatment with each indicated concentration. Fifteen were used per concentration (*n* = 15). One-way analysis of variance (ANOVA) was used to compare the differences between the average of the imaged areas between groups. *p < 0.05, **p < 0.01, and ***p < 0.00.1

Next, we wanted to determine the no observed effect concentration (NOEC), which is the highest concentration of the BNrGOTi nanoparticles that do not cause significant teratogenicity to zebrafish embryos in comparison to the negative control (Fig. 11B). These deformities (yolk and hear edema and short eye and body size) were present in the DEAB positive control (Fig. 11A). Analysis of the body (Fig. 11C), yolk (Fig. 11D), and eye (Fig. 11E) size results showed that the NOEC for the BNrGOTi nanoparticles that do not cause any abnormalities were between 150 and 200 mg L^−1^. All concentrations above 200 cause significant mortality (26.6%) to zebrafish embryos (Fig. 10A). In order to confirm the results of the NOEC concentration for the BNrGOTi nanoparticles, further analysis was carried out on the 0, 50, and 150 mg L^−1^ concentrations. Cardiotoxicity analysis showed that there was no significant difference in the dorsal aorta (DA) and the pericardial vein (PCV) heart rate between all the BNrGOTi nanoparticle concentration-treated (0, 50, and 150 mg L^−1^) embryos and the negative control, suggesting that the NOEC for the BNrGOTi nanoparticle was 150 mg L^−1^. These results provide another line of evidence that BNrGOTi nanoparticles are eco-friendly photocatalysts.

## Conclusions

BNrGOTi nanocomposites of different concentrations were effectively synthesized through a hydrothermal method. The prepared composites were characterized using different surface and bulk techniques. The study revealed that a composite of 10% of BN and 0.1 % rGO loaded on commercially P25 TiO_2_ is the best in terms of the phenol degradation percentage in the presence of H_2_O_2_. It showed that 93.4% of phenol degradation was acquired in 30 min using a Xe illumination as a sunlight simulator. Around 92% of the 93.4% of the degraded phenol was completely mineralized, as was revealed by the TOC measurements. The composite showed excellent durability. Besides, it is also an eco-friendly photocatalyst, as was proved from the ecotoxicological assessment using the zebrafish embryo model.

## Data Availability

The datasets used and/or analyzed during the current study are available from the corresponding author on reasonable request.
